# The effect of balance training on ankle proprioception in patients with functional ankle instability

**DOI:** 10.1186/1757-1146-7-S1-A37

**Published:** 2014-04-08

**Authors:** Tarang K Jain, Clayton N Wauneka, Wen Liu

**Affiliations:** 1 Physical Therapy and Rehabilitation Science, University of Kansas Medical Center, Kansas City, KS, 66160, USA; 2 Bioengineering Graduate Program, University of Kansas, Lawrence, KS, 66405, USA

## Background

Approximately 40-70% of individuals who suffer an ankle sprain report residual symptoms 6 weeks to 18 months post injury [1]. Balance training is often the first choice of treatment in patients with functional ankle instability (FAI); however the effect of balance training on the ankle proprioceptive sensation in these patients is debatable [2].

## Purpose

To examine the effect of 4-week balance training intervention on self-reported ankle instability using Cumberland ankle instability tool questionnaire (CAIT) and ankle joint position sense (JPS) using joint position-reposition test in patients with FAI.

## Methods

Twenty-four recreationally active patients with unilateral FAI were randomized to either the control (n = 12, 34.6±9.04 years, CAIT score = 13.9±4.3) or experimental (n = 12, 33.8±6.4 years, CAIT score = 13.4±3.3) group. Patients in the experimental group were trained on the affected limb using static and dynamic balance components with Biodex balance stability system. CAIT questionnaire was administered at baseline and 6-week post-intervention. The passive ankle JPS at 15 and 30 degrees of ankle inversion on the affected and unaffected limbs was measured at baseline and 4-week post-intervention using Biodex dynamometer. CAIT questionnaire score and mean error in angular displacement at baseline and post-intervention were compared using two-tailed paired Student t tests.

## Results

**Figure 1:**
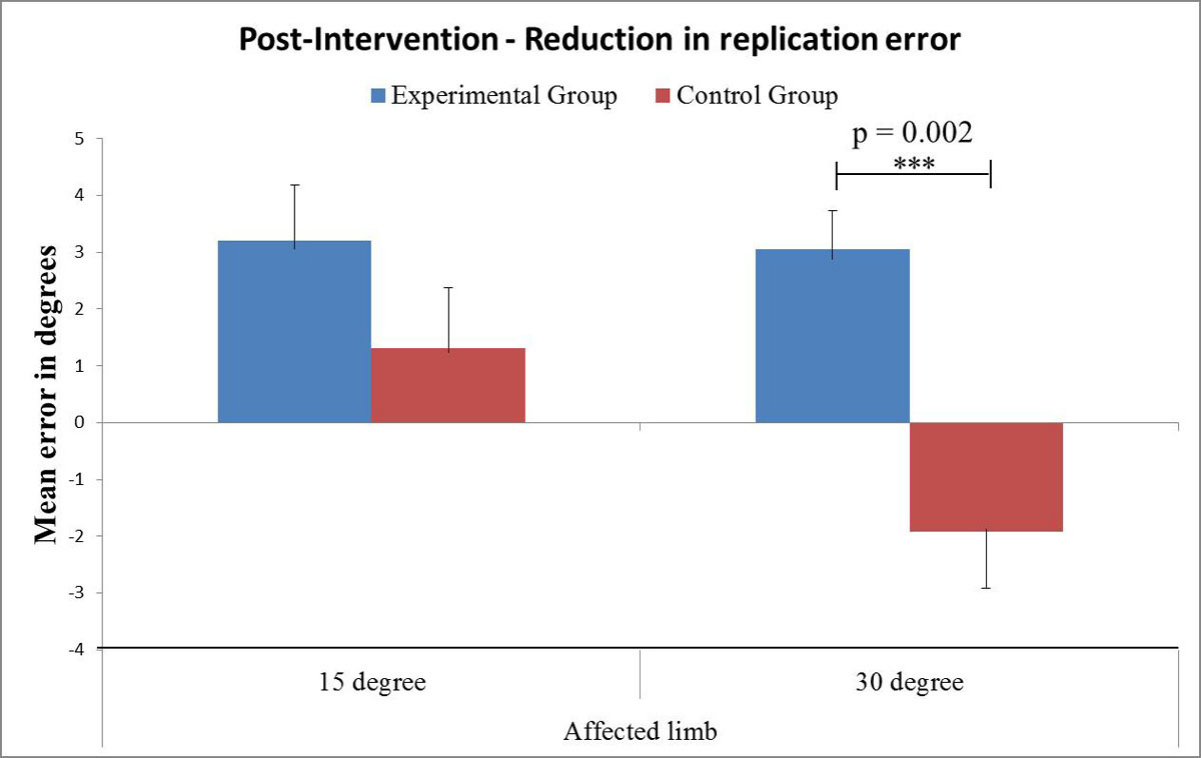
Reduction in mean replication error in both the groups following balance training intervention

At baseline, CAIT questionnaire scores were similar between the two groups. There was a significant side-to-side difference in the mean error at 30° (4.1±2.6 vs. 2.5±2.0, p=0.03, 95% CI [0.170, 3.024]) of ankle inversion. Following balance training, the experimental group showed significant improvement in CAIT questionnaire score (22.3±2.5, p=0.001, 95% CI [2.983, 9.183]). The experimental group also showed significant reduction in mean error on the affected limb following intervention at both 15° (1.9±1.4, p = 0.008, 95% CI [-5.376, -1.013]) and 30° (1.4±1.2, p = 0.001, 95% CI [-4.531, -1.580]) of ankle inversion. When compared to the affected limb in the control group, affected limb in the experimental group demonstrated significant reduction in mean error at 30° (p=0.002) but not at 15° of ankle inversion following balance training intervention (Figure 1).

## Conclusion

The 4-week balance training program was effective in reducing the self-reported ankle instability and improving the deficit of ankle joint position sense in patients with FAI.

Level of evidence: Therapy, 2b

ClinicalTrials.gov Identifier: NCT00703456

Supported by NIH Grant R21 AR062205 and Kansas Partners in Progress, Inc.
